# Vitiligo as the Presenting Manifestation of Sjogren’s Syndrome: Case Report and Review of Vitiligo and Its Associated Autoimmune Conditions

**DOI:** 10.7759/cureus.11250

**Published:** 2020-10-29

**Authors:** Nikolas Gutierrez, Philip R Cohen

**Affiliations:** 1 General Practice, 1st Marine Division, 1st Combat Engineer Battalion, Camp Pendleton, USA; 2 Dermatology, San Diego Family Dermatology, National City, USA

**Keywords:** autoimmune, hypopigmentation, sjogren’s syndrome, vitiligo, xeropthalmia

## Abstract

Vitiligo is a cutaneous condition that causes loss of pigmentation; it can be associated with other autoimmune conditions. Sjogren’s syndrome is a chronic inflammatory autoimmune condition that classically presents with xerophthalmia and xerostomia. Rarely, vitiligo has occurred in individuals with Sjogren’s syndrome. A 51-year-old Hispanic woman presented with vitiligo. Her laboratory investigation was consistent with Sjogren’s syndrome; she subsequentially developed xerophthalmia and arthritis. The association between vitiligo and other autoimmune conditions is reviewed and the association of vitiligo and Sjogren’s syndrome is discussed.

## Introduction

Vitiligo is an autoimmune condition in which there is loss of skin pigmentation. The pathogenesis of vitiligo is poorly understood but has been postulated to be caused by inherent defects in intracellular oxidative stress mechanisms and autoantibodies targeting melanocytes. Several therapeutic modalities, of varying efficacy, may be initiated to attempt to provide repigmentation [1].

Sjogren’s syndrome is a chronic inflammatory autoimmune condition characterized by key features such a dry mouth (xerostomia) and dry eyes (xerophthalmia) [2]. Laboratory findings include a positive antinuclear antibody (ANA) titer as well as antibodies to Sjogren’s syndrome A (SS-A/Ro) and/or Sjogren’s syndrome B (SS-B/La) [3]. Cutaneous manifestations of Sjogren’s syndrome are infrequent; however, in addition to vitiligo, they may include alopecia, dryness of the skin, purpura, and vasculitis presenting morphologically similar to urticaria [2,4].

A 51-year-old Hispanic woman who presented with vitiligo and subsequently developed clinical features of Sjogren’s syndrome is described. Autoimmune conditions associated with vitiligo are reviewed. Also, the association between Sjogren’s syndrome and vitiligo is discussed.

## Case presentation

A 51-year-old Hispanic woman presented for evaluation of progressive loss of pigmentation on her chest, abdomen, and bilateral flanks. She first noticed the hypopigmented patches two years earlier after a severe sunburn at the beach. Her past medical history is notable for hypothyroidism which was diagnosed 26 years earlier and managed with levothyroxine 75 mcg daily. 

**Figure 1:**
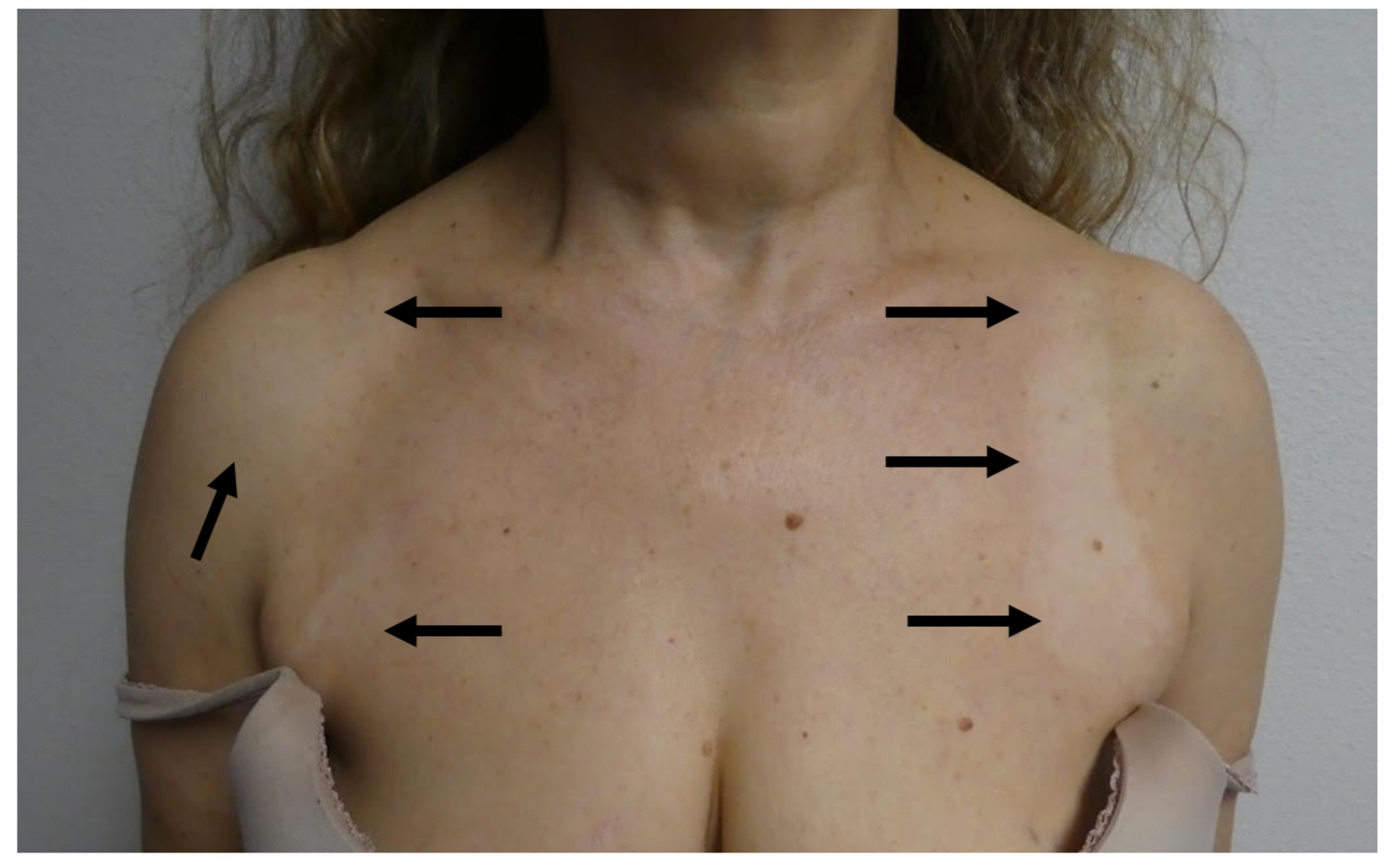
The initial clinical presentation of vitiligo as hypopigmented patches on anterior chest following a sunburn A 51-year-old woman whose initial manifestation of Sjogren’s syndrome was vitiligo that appeared two years earlier after she experienced a severe sunburn. Hypopigmented patches (black arrows) are present on her chest.

**Figure 2:**
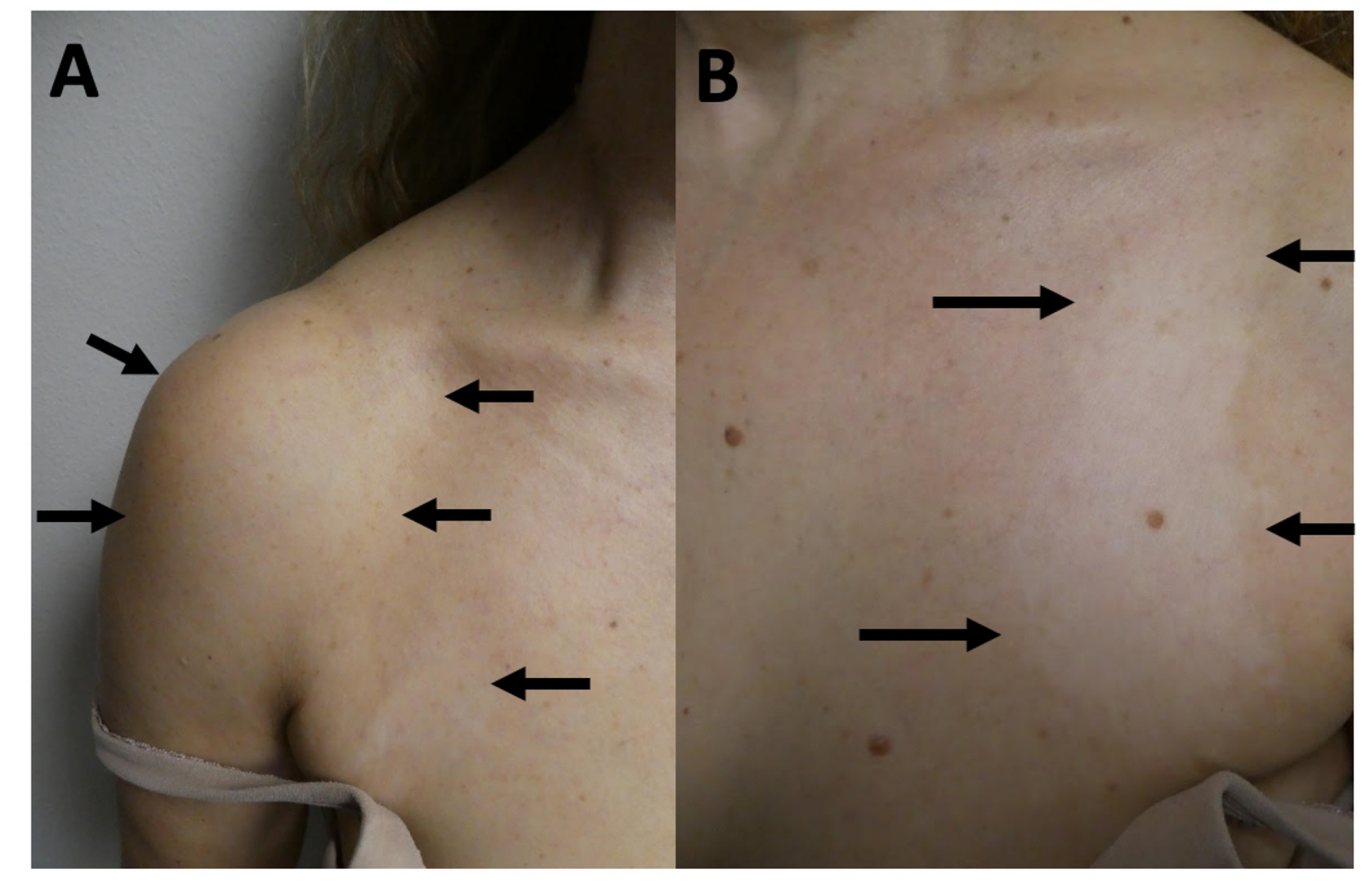
Sjogren’s syndrome presenting as vitiligo Closer views of the hypopigmented patches (black arrows) of vitiligo on the right anterior shoulder and chest (A) and the left side of the chest (B).

**Figure 3:**
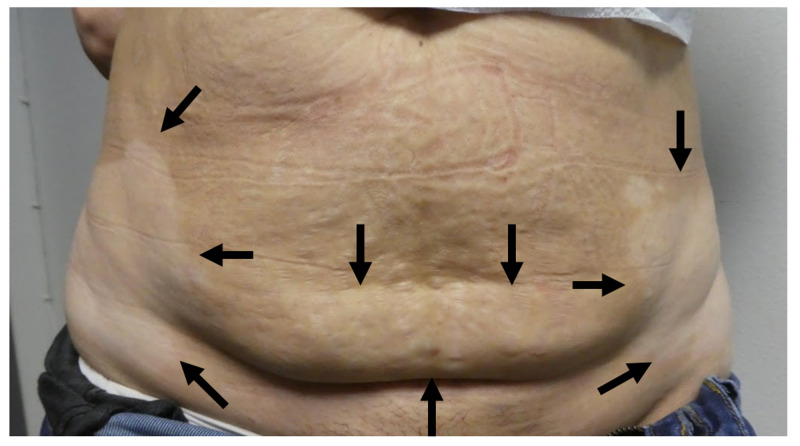
Vitiligo as the presenting manifestation of Sjogren’s syndrome A 51-year-old woman with vitiligo that appears as hypopigmented patches (black arrows) on the abdomen and bilateral flanks.

**Figure 4:**
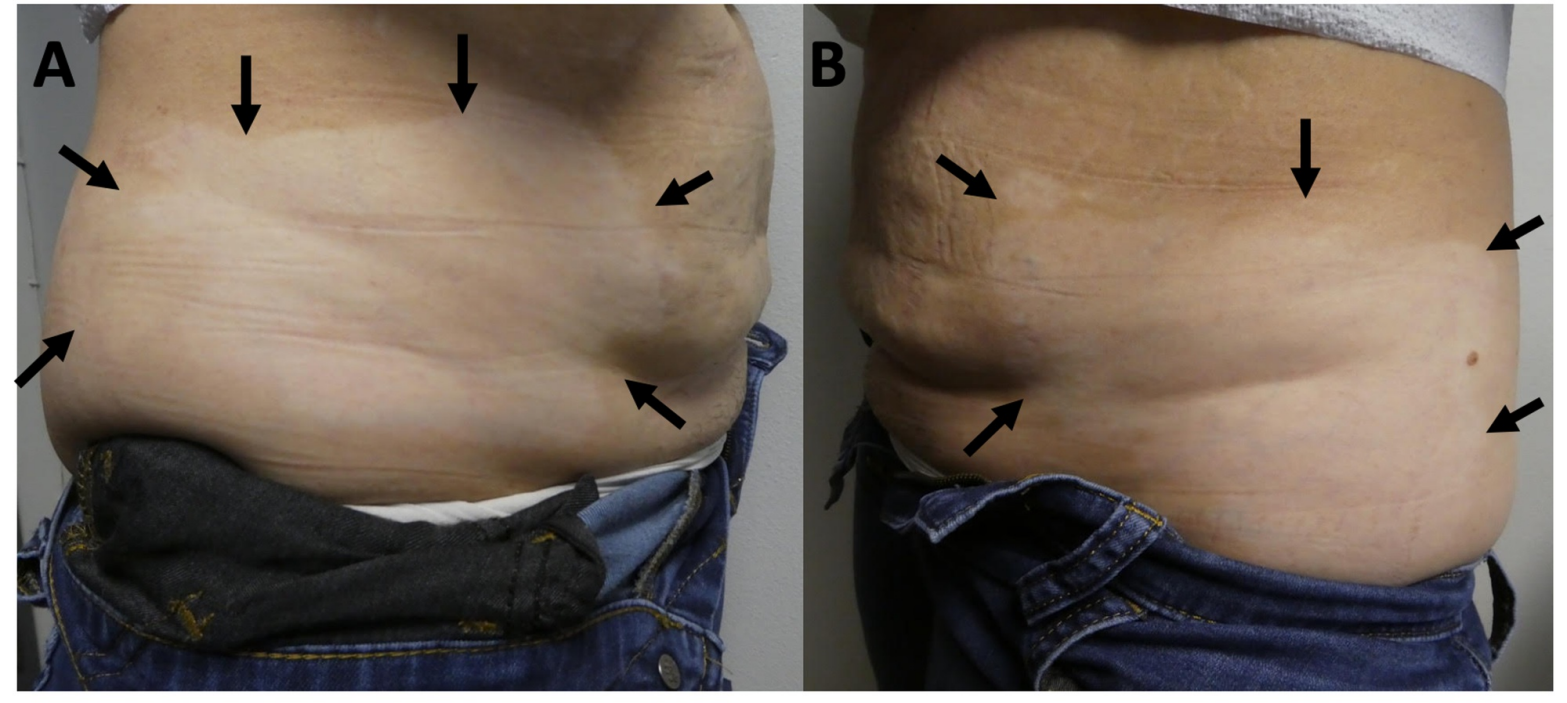
Sjogren’s syndrome presenting as vitiligo Closer views of hypopigmented patches (black arrows) of vitiligo on the right flank (A) and the left flank (B); the vitiligo extends to her lower back.

Physical examination revealed hypopigmentation on her chest extending from the anterior shoulders to the breasts bilaterally (Figures 1, 2). The absence of pigment was also noted on her lower abdomen from the umbilicus to the suprapubic region and extended bilaterally to involve the flanks and lower back (Figures 3, 4). There was no periocular, perioral, vulvar, perianal, or distal extremity hypopigmentation. 

Physical examination established the diagnosis of vitiligo. Laboratory evaluation for systemic diseases potentially associated with vitiligo was performed; the studies included: complete blood count (CBC), complete metabolic panel, ANA titer, rheumatoid factor (RF), double stranded-DNA (ds-DNA) antibody, Smith (Sm) antibody, ribonucleic protein (RNP) antibody, chromatin antibody, SS-A/SS-B antibodies, anti-topoisomerase I (scl-70), Jo-1 antibody, folate, vitamin B12, and thyroid-stimulating hormone (TSH). Laboratory results were notable for a positive ANA titer (1:80; normal, <1:80) with a homogenous pattern and an elevated SS-B/La antigen (4.3 AI; normal, < 0.9 AI). The remainder of her laboratory work-up was unremarkable. 

Correlation of her clinical presentation and the laboratory studies suggested the possibility of Sjogren’s syndrome. Within three months of her initial evaluation, she had developed dry eyes and arthritis; however, she did not have a dry mouth.

Initial management of her vitiligo included twice daily application of topical corticosteroid cream (triamcinolone 0.1%) which irritated her skin. Therefore, the treatment was discontinued. Thereafter, she declined further topical therapy, refusing alternative corticosteroids and calcineurin inhibitors, as well as phototherapy. 

During the subsequent 12 months of observation, her primary hypopigmented patches remained unchanged. However, she did develop a new patch of hypopigmentation on the medial aspect of her right upper arm. She maintains a regular follow-up with her dermatologist every six months.

In addition to the dermatologic evaluation, she has also been evaluated by both a rheumatologist and an ophthalmologist. The rheumatologist recommended daily hydroxychloroquine, but she refused treatment. The ophthalmologist’s initial evaluation confirmed the presence of xerophthalmia; symptomatic management of her dry eyes with carboxymethylcellulose artificial tears at least twice daily was initiated. She maintains regular follow-up with both the rheumatologist and ophthalmologist every six months.

## Discussion

Vitiligo is an autoimmune condition in which there is a loss of melanocytes in the skin and mucus membranes. It affects between 0.5% to 2% of the world’s population without a specific race or sex predilection [5]. The onset of vitiligo typically occurs during the ages of active growth; 50% of cases occur before the age of 20 years and 70%-80% of cases occur prior to the age of 30 years [6].

The pathogenesis of vitiligo is unknown. However, it has been postulated that the relationship between environmental factors and genetic predisposition results in the destruction of melanocytes in patients with vitiligo. In these individuals, numerous genetic variants in the innate and adaptive immune system have been identified; these observations are supportive of the autoimmune theory of vitiligo pathogenesis [4]. 

There are also other theories for the pathogenesis of vitiligo. They include the adhesion defect theory in which there is chronic detachment of melanocytes due to trauma and a biochemical theory in which alterations in redox balance occurs and results in melanocyte destruction. Whether an environmental trigger, such as a trauma, or an inherent immunologic variant is the initial event resulting in the destruction of melanocytes remains to be determined [7,8]. 

Clinical variants of vitiligo include focal, nonsegmental, segmental, and mixed. Nonsegmental subtypes include generalized (symmetric, bilateral hypopigmented patches), acral (only extremities), acrofacial (extremities and face), mucosal, and universal (greater than 80% of body surface area). Segmental vitiligo is unilateral, asymmetric hypopigmented macules that lie within a dermatomal distribution. The mixed variant initially presents as segmental vitiligo that progresses to nonsegmental vitiligo [5,7].

There are many treatment options - each with varying efficacy - for vitiligo. Topical corticosteroids, topical calcineurin inhibitors, and/or phototherapy are typically initiated as first-line measures due to the ease of access, low cost, and efficacy [7,8]. As the pathogenesis of vitiligo is further elucidated, immunomodulators are emerging as potential treatment modalities. However, further research and testing are being conducted prior to the widespread implementation of these agents [1,8].

Autoimmune diseases, such as vitiligo, may initially present as a solitary condition; however, there is an increased likelihood of subsequently developing another autoimmune condition. In previous studies, 14-55% of patients with vitiligo were found to have comorbid autoimmune conditions. Associated autoimmune conditions include inflammatory bowel disease, pernicious anemia, systemic lupus erythematosus, thyroid disease, and less commonly, Sjogren’s syndrome (Table 1) [9-11].

**Table 1:** List of common vitiligo-associated autoimmune conditions

Condition	References
Alopecia areata	[10,11]
Atopic dermatitis	[10,11]
Diabetes mellitus (type I)	[10,11]
Grave’s Disease	[9-11]
Hashimoto’s Thyroiditis	[9-11]
Idiopathic Thrombocytopenic Purpura	[9-11]
Inflammatory Bowel Disease	[9-11]
Multiple sclerosis	[9-11]
Myasthenia Gravis	[9-11]
Pernicious Anemia	[9-11]
Psoriasis	[10,11]
Rheumatoid Arthritis	[9-11]
Sjogren’s syndrome	[10,11]
Systemic Lupus Erythematous	[9-11]

Sjogren’s syndrome is a chronic condition with an estimated prevalence of 0.1% to 0.4%. It primarily affects lacrimal and salivary glands. Most patients present with dry eyes and/or dry mouth [2,11,12]. 

Extraglandular manifestations of Sjogren’s syndrome can include arthralgias, anemia, cytopenia, hypogammaglobinemia, lung disease, lymphoma, and myalgia. Skin manifestations associated with Sjogren’s syndrome are uncommon and are often under-reported. Cutaneous features of Sjogren’s syndrome include alopecia, purpura, urticaria-like vasculitis, vitiligo, and xerosis [2,12]. 

The pathogenesis of Sjogren’s syndrome is thought to be initiated by a trigger--either environmental, hormonal, or viral--that activates toll-like receptors on salivary gland epithelial cells, initiating a cascade of events lead by interferons and cytokines that result in chronic autoimmune epithelitis in genetically susceptible patients. Anti-Ro and anti-La have been found in salivary gland cells and they cause apoptosis; furthermore, anti-Ro is strongly suspected to have a pathogenetic role in the development of extraglandular manifestations of Sjogren’s syndrome. Hence, the etiology of Sjogren’s syndrome is likely to be multifactorial and involve not only epigenetics but also environmental factors [3].

Patients with vitiligo have been observed to subsequently develop Sjogren’s syndrome. In a cross-sectional study of comorbid autoimmune diseases in patients with vitiligo, Gill et. al. observed a 13-fold increase in the prevalence of Sjogren’s syndrome in vitiligo patients [11]. In addition, a retrospective population-based cohort study by Chen et. al found a statistically significant association of vitiligo and Sjogren’s syndrome in older female patients [10].

Our patient presented with vitiligo following a severe sunburn two years earlier. Laboratory evaluation was performed to screen for additional vitiligo-associated autoimmune disorders. A positive ANA titer and antibodies to SS-B were discovered. 

A positive ANA titer is a non-specific marker for autoimmune disease. As an isolated finding, it is not diagnostic of disease. However, a positive ANA titer along with the presence of other antibodies can be associated with systemic lupus erythematosus, scleroderma, and Sjogren’s syndrome. 

Conversely, the presence of an autoantibody to SS-B is highly suggestive of Sjogren’s syndrome. Though our patient did not initially present with classic symptoms of Sjogren’s syndrome, she subsequently developed dry eyes and arthritis three months after her initial evaluation. Her prior - though remote - history of hypothyroidism may also be related to her vitiligo, Sjogren’s syndrome, or both; however, since her diagnosis of thyroid disease was established 26 years earlier, we favor that the vitiligo was the presenting manifestation of her Sjogren’s syndrome. 

Although the lesions of vitiligo are isolated to the skin, this condition can be associated with other autoimmune conditions. Therefore, it is reasonable for the clinician to evaluate their patient for vitiligo-associated diseases. The laboratory studies to consider for evaluation may include- but are not limited to- the following: ANA titer, anti-dsDNA antibody, anti-RNP antibody, anti-Scl 70 antibody, CBC, folate, RF, SS-A/SS-B antibodies, thyroid function panel (TSH, thyroxine, and triiodothyronine), thyroid antibodies (anti-microsomal antibody, anti-peroxidase antibody, and anti-thyroglobulin antibody), vitamin B12, and serum chemistries including glucose and hemoglobin A1c.

## Conclusions

Autoimmune diseases can occur as solitary conditions or multiple disorders either concurrently or sequentially. Vitiligo is a cutaneous manifestation of Sjogren’s syndrome; however, it is uncommon and infrequently described in Sjogren’s syndrome patients. We describe a woman who developed vitiligo as the initial presentation of Sjogren’s syndrome. Laboratory studies for associated autoimmune syndromes, including Sjogren’s syndrome, should be considered in a patient with the new onset of vitiligo.
